# Luminescence Sensors Applied to Water Analysis of Organic Pollutants—An Update

**DOI:** 10.3390/s111211081

**Published:** 2011-11-28

**Authors:** Gabriela A. Ibañez, Graciela M. Escandar

**Affiliations:** Instituto de Química Rosario (CONICET-UNR), Facultad de Ciencias Bioquímicas y Farmacéuticas, Universidad Nacional de Rosario, Suipacha 531 (2000) Rosario, Argentina; E-Mail: ibanez@iquir-conicet.gov.ar

**Keywords:** luminescence, sensors, pollutants, environment

## Abstract

The development of chemical sensors for environmental analysis based on fluorescence, phosphorescence and chemiluminescence signals continues to be a dynamic topic within the sensor field. This review covers the fundamentals of this type of sensors, and an update on recent works devoted to quantifying organic pollutants in environmental waters, focusing on advances since about 2005. Among the wide variety of these contaminants, special attention has been paid polycyclic aromatic hydrocarbons, pesticides, explosives and emerging organic pollutants. The potential of coupling optical sensors with multivariate calibration methods in order to improve the selectivity is also discussed.

## 1. Introduction

The imperative necessity of simple and reliable analytical techniques in areas such as industry, pharmacy, the environment and medicine has produced a significant impulse for the development of novel chemical sensors. The relevance of applying chemical sensors to environmental measurements lies in the many advantages these devices offer, such as online detection, miniaturization enabling at-site measurements and minimal waste production, which contributes to green chemistry. In the present review we include selected works devoted to the development of both sensors themselves and probes for the study of organic pollutants in environmental waters since 2005.

It should be noted that some of the literature terminology related to sensor is ambiguous. A chemical sensor is commonly defined as a device that, as the result of its chemical interaction with an analyte, transforms either qualitative or quantitative chemical information into an analytically useful signal [1]. A sensor was also defined as a molecule (or nanoparticle) acting as a reporter moiety that communicates the presence of an analyte via modulation of an analytical signal [2,3]. Strictly speaking, a chemical sensor is a miniaturized device capable of providing continuous real-time and on-line information about the presence of specific analyte(s) in a sample [4,5]. However, as indicated above, it is common to consider sensors as probes which, although of interest for designing finished sensors, only represent a component of them. In other words, while sensing would refer to a continuous process, probing would refer to single-shot testing [4].

Chemical sensors are classified according to different criteria [6]; a usual one is the type of analytical signal measured. Although a significant variety of sensors have been developed, those based on either electrochemical or optical signals are the most popular [7]. Absorbance, reflectance, Raman dispersion or luminescence emission (fluorescence, phosphorescence and chemiluminescence) signals are involved in optical sensors.

Commonly, in luminescence sensors the emitted fluorescence, phosphorescence or chemiluminescence signals are measured after the analyte is immobilized in a suitable solid support, giving origin to the expression solid-phase luminescence (SPL) or to its equivalent solid-matrix luminescence (SML). Under certain conditions, these analytical signals can be related to the concentration of analyte in the sample and a quantitative analysis is performed.

Biannual reviews devoted to molecular luminescence spectrometry, which included advances in sensors and solid-surface luminescence methods, have been published by Warner’s research group [2,8,9]. Molina Díaz *et al*. [10] and recently Ruiz Medina *et al.* [11] have published comprehensive reviews of the basic principles and practical considerations of optosensors. Bosch Ojeda and Sánchez Rojas reviewed methods involving optical sensors coupled to flow injection analysis for environmental, biomedical and industrial samples [12] and, more recently, Wang *et al*. discussed the development of flow-based luminescence sensors for environmental water analysis, including selected organic and inorganic contaminants [13]. Very recently, Woutersen *et al.* have reviewed the use of biosensors based on luminescent bacteria as valuable tools to monitor the presence of organic pollutants, heavy metals and other compounds in drinking waters [14].

In most probe configurations (Table 1) the analyte is retained by adsorption, binding or entrapment, either on a plane surface (via direct deposit or through an extraction procedure with a syringe) [15–17], or by immobilizing it in microbeads which are then transferred to a measuring cell before each determination [6,18–20]. Common plane surface supports are filter-papers and membranes of different materials such as C_18_-disks, nylon membranes and polymeric membranes. On the other hand, microbeads are constituted by polymeric materials, ion-exchange resins, bonded-phase silicagel, *etc*. Working with microbead configurations requires certain operator skills and has the disadvantage of involving longer analysis time *per* sample. Therefore, this technique is being replaced by flow methodologies.

The signal measured in optical probes is produced by either the analyte itself (due to some intrinsic optical property) or by an immobilized indicator able to interact with the analyte and indirectly test its presence. As will be discussed below, luminescence probes have been significantly exploited for the detection and quantification of organic pollutants, but the developed methods are in general limited by the low sampling frequency.

The usefulness of an SPL probe is increased significantly when it is coupled to flow-systems, yielding sensors which combine the retention of the analyte (or its reaction product) in active solid supports with on-line luminescence detection. Thus, the advantages of sensitivity and relative selectivity of SPL are added to those corresponding to methods carried out in flow systems, which are mainly speed, automation and the possibility of implementing on-line monitoring procedures. Techniques used for performing luminescence sensor methodologies involve flow-injection analysis (FIA) [18,21], sequential-injection analysis (SIA) [22,23], multi-syringe flow-injection analysis (MSFIA) [18,24,25], bead-injection spectroscopy (BIS) [26,27], and multi-commutation [18,28–30].

The most common solid supports used as sensor zones for packing the flow cells are C_18_ bonded phase silica beads, ion-exchange resins, and polymers without exchangeable groups [10]. Nylon powder was recently introduced as a novel material for this purpose (see below) [31,32].

**Figure 1.**
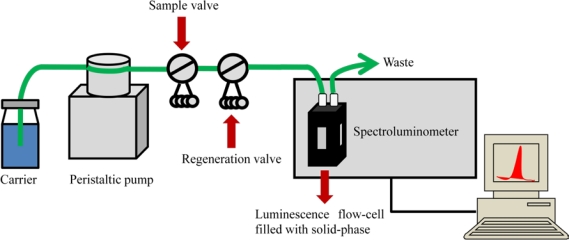
Schematic diagram of a flow-through optosensor system.

In most flow-through optosensors the same solid-phase placed in the cell is successively reused a number of times (Figure 1). Thus, an important amount of supplies and material are saved, and the experimental time involved in each determination significantly decreases in comparison with batch methodologies.

**Figure 2.**
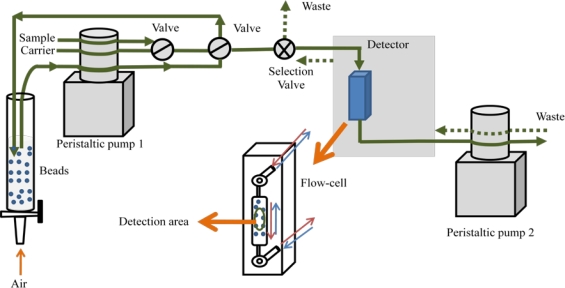
Bead injection manifold.

A working alternative for cases where either the retained analyte cannot be efficiently removed, or malfunctions of the sorbent surface occur between subsequent measurements, is the use of the bead-injection technique [26,27]. This scheme is based on a renewable surface sensing for each measurement in flowing systems (Figure 2). After each run, the support is discarded by flow reversal and the sensor surface is renewed by injecting a new plug of a fresh bead suspension. Thus, the problems mentioned above are easily overcome.

The sensor zone for luminescence sensor methodologies can also be constituted by fluorophores, dyes or enzymes immobilized through adsorption or covalent bond in thin polymer membranes or controlled-pore glass for either the direct or indirect determination of the analyte of interest [6,12,33]. Recently, fluorescent conjugated polymers were combined with quantum dots (QDs) for sensing inorganic ions and biochemical species [34], and their incorporation to molecular imprinting polymers (MIPs, see below) is planned in future research.

QDs and metal and silica nanoparticles are starting to replace traditional organic molecules as detection labels because they offer superior optical properties [35,36]. However, although some applications in environmental matrices have been reported (see below), they have been mainly focused to the analysis of biological samples.

Materials obtained from the sol-gel process have demonstrated to be suitable for the development of optical sensors [37]. These solid supports are chemically inert, possess low intrinsic luminescence and a high rigidity, and are both photochemically and thermally stable [38].

It is common that optical sensing configurations, either as a probe-type or as a flow-through cell sensor, involve the use of optical fibers [39]. An optical fiber can transmit the electromagnetic radiation to and from a sensing region which is in direct contact with the sample [37]. The most frequent design for sensors involving optical fibers is a distal-type sensor in which a sensor membrane is immobilized either at the end of the optical fiber, or along a section of its core [37]. The chemical transducer can be either directly deposited in the fiber or encapsulated in a polymeric matrix (e.g., sol-gel thin film). Optical-fiber sensors based on sol-gel films have been intensely explored as pH, gas, ionic species and solvent sensors. Doong *et al*. demonstrated the ability of a sol-gel encapsulated acetylcholinesterase fiber optic biosensor to carry out the analysis of the organophosphorus pesticide paraoxon in spiked samples [40]. Although the sensor was not applied to real samples, its potential utility was indicated.

A relevant disadvantage of the SPL methodology with most of the above mentioned sensor supports is the lack of selectivity when multicomponent samples are investigated, due to the probability that similar compounds show similar luminescence properties. The use of MIPs as solid-supports is a good option for improving the selectivity of an optosensor method [41–43]. Molecular imprinting technology is based on the co-polymerization of appropriate monomers in the presence of a target molecule (the analyte itself or a molecule with a very closely related structure), which acts as a molecular template. The imprinted molecule is then removed by a washing step and the resulting material contains cavities which are a frozen print of the original template molecule employed. Thus, the polymer would be able of selectively rebinding the analyte through a selective recognition.

With the aim to obtain suitable optical sensing phases, different strategies for the preparation of magnetic MIPs for pyrene as model were recently evaluated [44]. The incorporation of magnetic properties is useful for implementing optical sensing phases in portable devices to control analytes of interest in matrices such as water, solvents, *etc*.

However, while the MIP approach is suitable for a single component, it is not appropriate for the simultaneous determination of several analytes in a sample. Further, the difficulties grow if interferents are present. A useful way to resolve the spectral overlapping in complex matrices is the incorporation of multivariate calibration methods. Indeed, the latter is one of the most widely used strategies for ensuring interference-free quantitation in environmental analysis [45]. Specifically, some second-order calibration methods achieve the so called ‘second-order advantage’, which allows the quantitation of analytes even in the presence of unexpected sample constituents [46].

Valero Navarro *et al*. [47] reported the first application of the second-order advantage to a fluorescence optosensor, determining two naphthylamines in drinking waters in the presence of 1-naphthalenemethylamine (as interference) using a rather selective MIP-fluorescence optosensing system. Then, the potential of the second-order advantage was evaluated in the simultaneous determination of two fungicides (thiabendazole and fuberidazole), using an unspecific supporting material (C_18_-bonded phase) and in the presence of three real interferences (carbaryl, carbendazim and 1-naphthylacetic acid) [48].

In the following sections, the material was organized paying attention to recent optosensors for determining concerned compounds due to their deleterious effects to the ecosystem and to humans, namely polycyclic aromatic hydrocarbons (PAHs), agrochemicals, explosives, and other contaminants.

## 2. Polycyclic Aromatic Hydrocarbons (PAHs)

PAHs (organic compounds formed by fused aromatic rings with no heteroatoms or substituents) are very toxic pollutants to aquatic life and suspected to be human carcinogens. The first problem to be faced when PAHs are determined in natural water samples is the potentially very low concentration levels, because of their low water solubilities [49]. The United States Environmental Protection Agency (US-EPA) has established in drinking water values of 0 and 200 ng L^−1^ for the maximum contaminant level goal and the maximum contaminant level, respectively, for benzo[*a*]pyrene (BaP), one of the most carcinogenic PAHs [50]. On the other hand, the European Union (EU) and the World Health Organization (WHO) have established a maximum admissible concentration for BaP of 10 ng L^−1^ in water intended for human consumption [51]. In any case, it is evident that very sensitive sensors must be developed to achieve a successful result. A second problem related to luminescence sensors for these compounds is that many PAHs exhibit similar luminescence properties, highlighting the need of a significant selectivity in such methodologies.

A flow-through phosphorescence optosensor, based on a non-ionic resin, was optimized to quantify BaP in the presence of other PAHs in water samples. The optosensor showed a detection limit of 12 ng mL^−1^, a relative standard deviation of 5% at 50 ng mL^−1^ of BaP, and a response time of 315 s [ 52]

Trace amounts of fluoranthene in water were determined phosphorimetrically using an MIP containing an internal heavy atom in its polymeric structure [53]. The implemented flow-through optosensor demonstrated a high specificity against other PAHs and an LOD of 35 ng L^−1^ was achieved using 5 mL of sample.

Nylon has been shown to be an excellent probe material for PAHs, especially when a solid-phase extraction on a 6,6-nylon membrane via a syringe procedure is carried out on an aqueous solution of PAHs [54]. The primary chemical structure of nylon consists of amide groups separated by methylene sequences. The amide group is basically planar, due to the partial double-bond character of the C–N bond, and the chains are oriented in such a way as to maximize hydrogen bonding between the amino and carbonyl groups. One of the factors leading to a successful PAH extraction is the use of an aqueous phase to carry the hydrophobic analyte through the nylon membrane. While nonpolar interactions are produced between PAHs and the methylene nylon chains, the hydrophilic amide groups are expected to enhance the water movement into the sorbent, improving mass transfer and making it more effective.

This ability of nylon was exploited for the determination of BaP, measuring its fluorescence or phosphorescence signals over the surface after the extraction of 10–50 mL of different types of natural waters [54]. The analytical figures of merit obtained under the best experimental conditions demonstrated the capability of detecting BaP at a sub-parts-per-trillion (sub-ng L^−1^) level.

Using a similar procedure, but coupling the fluorescence measurement with second-order multivariate calibration, BaP and dibenz[*a*,*h*]anthracene (DBA) were determined at parts-per-trillion levels in a very interfering environment [55].

Very recently, a flow-through optosensor, using silica gel C_18_-bonded phase as support, and interfaced to a fast-scanning spectrofluorimeter, was applied to the simultaneous determination of six heavy-PAHs, namely BaP, DBA, chrysene, benzo[*b*]fluoranthene, benzo[*k*]fluoranthene and benz[*a*]anthracene [56]. Second-order calibration with the U-PLS/RBL (unfolded partial least-squares/residual bilinearization) algorithm allowed the rapid determination in contaminated river water and activated sludges, competing very favorably with the reference CG-MS method.

## 3. Agrochemicals

Agrochemicals are defined as chemicals, such as fertilizers, hormones, pesticides (fungicides, insecticides, herbicides) which improve the production of crops. Many of these products represent a potential risk for human health and, therefore, worldwide government agencies have manifested their concern, establishing strict maximum limits depending on the corresponding toxicity. The European Union has set drinking water limits of 0.1 and 0.5 ng mL^−1^ for individual and total pesticides, respectively [57]. To comply with the legislation, sensitive analytical methods are required. Due to the high sensitivity of the photoluminescence sensors, they have been widely used to the determination of agrochemical residues. Applications of flow sensors in pesticide analysis, including those based on optical signals, have been very recently reviewed by Llorent Martínez *et al*. [58].

### 3.1. Fungicides

Most research works for residual fungicide detection involve gas and liquid chromatographies, including both conventional and mass spectrometric detection [59]. However, the general awareness of the need to develop methods derived from the principles of the green chemistry applied to samples of environmental interest suggests that the use of optosensor methodologies will increase in the future.

The coupling of multi-commutation and multi-optosensing was developed for the analysis of fuberidazole and *o*-phenylphenol in waters obtained from wells and rivers [60]. The same research group also resolved mixtures of 1-naphthol, *o*-phenylphenol and thiabendazole, coupling a first-order multivariate approach to a flow-through optosensor system [61]. The selected algorithm (PLS, partial least squares) allowed the mixture resolution, but did not resolve the problem of the presence of unexpected interferences in the samples.

Nylon membranes represented a satisfactory phosphorescence probe for thiabendazole [62], allowing its determination at low concentration levels in natural waters. The emission was improved through the presence of heavy-atoms and cyclodextrins, and the exclusion of molecular oxygen from the measuring cell.

Based on this ability of nylon membranes, it was investigated whether this capability was preserved in nylon powder when used as an optosensor packing. Nylon powder showed outstanding properties as a fluorescence optosensor for the determination of thiabendazole in tap, underground, mineral and river water samples [31]. Nylon powder also promoted room-temperature phosphorescence (RTP) from this fungicide, showing a superior selectivity in comparison with the fluorescent sensor [32]. In both optosensors, water was used as carrier to transport thiabendazole through nylon powder, avoiding large volumes of waste organic solvents usually involved in flow systems and decreasing the analysis time when real aqueous samples were analysed.

The simultaneous determination of carbendazim and thiabendazole was performed using fluorescence excitation-emission matrices obtained after the extraction of the analytes over a C_18_-membrane surface [63]. The ability of the PLS/RBL (partial least-squares with residual bilinearization) chemometric algorithm to overcome the problems caused by the presence of both inner-filter effects and unsuspected species was demonstrated in both artificial and real water samples.

The simultaneous determination of thiabendazole and fuberidazole at part-per-billion levels in river, underground, mineral and tap water samples was performed coupling a flow-through optosensor to second-order chemometric analysis [48]. Excitation-emission fluorescence matrices of the retained fungicides were directly read on the C_18_-bonded phase support and were processed by different algorithms. U-PLS/RBL allowed to reach selectivity using a commercial but non-selective sensing support. The sample frequency, including excitation/emission fluorescence matrix measurements, was 12 samples h^−1^.

### 3.2. Insecticides

Viveros *et al*. have described a fiber-optic fluorescent-based biosensor for the detection of organophosphate insecticides and chemical warfare agents [64]. The bio-recognition element was the enzyme organophosphate hydrolase, which was conjugated with carboxynaphthofluorescein (a fluorescent reporter) and anchored on the optical waveguide of a portable fluorimeter. Organophosphates were quantitatively detected in the range 1–800 μM. Imidacloprid was determined in river, well and irrigation waters by a flow-through optosensor based on photochemically induced fluorescence (PIF) [65]. The insecticide is derivatized on-line by irradiation with UV light, providing a fluorescent photoproduct which is retained in C_18_ silica gel placed in the flow-cell. The greatest advantage introduced by the developed optosensor is its high throughput and the low cost of reagents in comparison it with batch methods.

The insecticide pentachlorophenol was determined in water through the room temperature phosphorescence developed by anchoring an MIP layer on the surface of Mn-doped ZnS quantum dots via a surface molecular imprinting process [66].

### 3.3. Herbicides

An optical in-house sensor for herbicide detection was developed by Varsami *et al*. [67]. The sensor detects the chemiluminescence of the luminol/hydrogen peroxide reaction catalyzed by horseradish peroxidase (HRP), which can be disrupted by certain herbicides such as triazine, diazines, phenolic and urea derivatives. Both the photosystem II complex (thylakoid membranes extracted from higher plants) and the enzyme are immobilized on magnetic beads (the two active regions of the sensor), which are in turn magnetically entrapped. The water sample (pre-mixed with luminol) enters the fluidic channel, and is illuminated on the photosystem II region. The sample with the produced H_2_O_2_ flows towards the enzyme region where the chemiluminescence reaction takes place, and the light produced is detected through an optical fiber. The presence of herbicides in the thylakoid samples reduces the hydrogen peroxide measured in a concentration-dependent manner. The system combines the production and detection of hydrogen peroxide in a single flow assay by coupling the individual steps (a pump to force the sample mixed with luminol through the sensor, a light source (LED), and a detector module) in a portable device. Preliminary results obtained with real water matrices indicated that the biosensor could be satisfactory applied to real samples.

A biosensor based on immobilization of microalgal strains in a polycarbonate membrane was constructed for the detection of atrazine, simazine, diuron, isoproturon and paraquat, which affect algae photosynthesis, either increasing or decreasing the chlorophyll *a* fluorescence [68]. The biosensor allowed on-line measurements of aqueous solutions of the herbicides passing through a flow cell at concentrations between 0.05 and 500 ng mL^−1^ using chlorophyll fluorescence as the biosensor response signal.

Herranz *et al*. proposed a flow-through immunosensor for the analysis of simazine in fortified river waters [69]. The assay is based on a competitive immunoassay, where simazine (antigen) competes with a HRP-labeled simazine derivative for the sites of anti-simazine antibodies. The immunocomplexes are retained on an immunosorbent (controlled-pore glass covalently bound to protein A) packed in a reactor, and the amount of labeled antigen bound to the antibody is measured by fluorescence and is related to the antibody concentration in the sample. Although the method involves preparation of reagents, and atrazine and propazine (additional triazine herbicides) showed significant cross-reactivity, it has the advantage to be very sensitive, allowing measurements at nanograms per liter levels.

A flow-through optosensor based on PIF in a micellar medium was developed for the determination of metsulfuron-methyl in river, well and irrigation waters [70]. The micelles containing the UV generated photoproduct are strongly retained on C_18_ silica gel filling the flow-cell. The system showed a high throughput, about 35 samples h^−1^, depending on the injection volume.

Linuron was determined in both environmental and drinking water samples employing a flow-through PIF optosensor [71]. The presence of the surfactants in the sample, which modified the retention properties, was critical for the emission detection in the C_18_ silica support, being HTAC (hexadecyltrimethylammonium chloride) the surfactant that rendered better statistical results. Despite the sensitivity improvement provided by the optosensor, a preconcentration step had to be performed when linuron concentrations below 0.1 μg mL^−1^ were analyzed.

Isoproturon was analyzed in drinking water using a flow-through fluoroinmunosensor constituted by a sol-gel glass doped with antimonoclonal antibody placed in the flow cell [72]. A competitive assay between isoproturon in the water sample and labeled isoproturon takes place in the cell, and the fluorescence decrease as the analyte concentration increases is measured. Before the quantification, the analyte is selectively extracted on-line using a column with anti-isoproturon antibody encapsulated in a silica gel matrix. Alternatively, the extration and clean-up step can be accomplished off-line using a C18 cartridge. The on-line methods achieve a detection limit of 9.7 ng L^−1^.

### 3.4. Plant Growth Regulators

A flow-through-phosphorescence optosensor, using a non-ionic polymeric resin (Amberlite XAD-7) as sensor material, was developed for the quantitation of 1-naphthaleneacetic acid in both river and fountain water [73]. The analytical performance achieved by the method compared favorably with other reported RTP methodologies.

The same research group carried out the determination of 2-naphthoxyacetic acid using both fluorescence and phophorescence flow–through optosensors [74]. While Amberlite XAD-7 was used for the immobilization of the analyte in the fluorescence experiment, silica gel was used for the phosphorescence one. The advantages and disadvantages of each sensor were discussed, and although their predictive abilities were demonstrated in a sample of soil, they can apparently be applied for the determination of the plant growth regulator in other environmental samples.

### 3.5. Mixtures of Agrochemicals

Because different agrochemicals are often applied simultaneously for different purposes, many works are devoted to multiresidue determinations. The fungicides carbendazim and benomyl, and the very toxic insecticide carbofuran were determined in spiked environmental waters using an optosensor implemented with a previous separation of the analytes on a minicolumn placed just before the sensor. The latter was packed with the same solid support (C_18_ silica gel) as the flow-through cell [75]. Benomyl and carbofuran are firstly retained in the minicolumn, while carbendazim reaches the sensing material and its fluorescence transitory signal is read. Then, carbofuran and benomyl are successively eluted from the minicolumn using two different eluting solutions, and reach the sensing zone in a sequential mode. Limits of detection of 15, 35 and 68 ng mL^−1^ for carbendazim, benomyl and carbofuran, respectively, are obtained using 2 mL of sample.

A flow-through optosensor implemented with a PIF was reported for the simultaneous determination of the fungicide thiabendazole and the herbicide metsulfuron methyl in environmental waters [76]. Samples containing the pesticides in a micellar medium (sodium dodecyl sulfate) are injected in the carrier stream and flow through the photoreactor, where the UV light promotes the photodegradation of the herbicide into a fluorescent photoproduct, while thiabendazole does not suffer any significant degradation. Then, the metsulfuron methyl photoproduct and thiabendazole reach a minicolumn of C_18_-bonded phase silica gel, where only the former is strongly retained. Thiabendazole is eluted by the carrier itself, flowing up to the cell, also containing C_18_-bonded phase silica gel where it is transitorily retained and monitored. Then, the metsulfuron methyl photoproduct is desorbed with an eluting solution and carried to the detection area where it is retained and monitored.

## 4. Explosives and Related Compounds

Explosives are an important group of organic pollutants. In addition, they are produced in large quantities, and they may enter soil, air and water due to different activities with potential impacts on environmental and human health: explosive manufacture, assembly, packing and even detonation [77]. Singh reviewed the application of sensors, including optical ones, for the detection of explosives and related illicit materials [78].

Organophosphorus species used as chemical weapons (many of them chemically similar to insecticides) have been determined using an MIP sensor technique [79,80]. Detection through a miniature fiber-optic spectrometer is based on sensitized luminescence produced by selective binding between the analyte and a luminescent europium-containing reporter molecule included in the polymer.

An explosive of great concern is 2,4,6-trinitrotoluene (TNT), which can readily enter groundwater supplies, and has been classified as toxic at concentrations above 2 ng mL^−1^ by the Environmental Protection Agency [81]. Li *et al*. prepared a cross-linked molecularly imprinted fluorescent conjugated polymer for the detection of TNT and related nitroaromatic compounds [82]. Although the sensor was not applied to real samples, its intrinsic properties (mainly selectivity, stability and analytical response reversibility) it appeared to be valuable for potential chemosensing applications.

Gao *et al*. developed a method for detecting TNT in solution and vapor using resonance energy transfer-amplifying fluorescence quenching at the surface of silica nanoparticles as substrate [83].

A fluorescent-labeled imprinted polymer sensor (based on MIP microparticles prepared using methacrylic acid, and combined with fluorescent quantum dots) was developed to detect aqueous concentrations of TNT and 2,4-dinitrotoluene (DNT) [84]. Although the attained limits of detection are not low enough (approximately 0.5 and 1 mg L^−1^ for TNT and DNT, respectively), future optimizations have been proposed by the authors in order to improve the method sensitivity.

A fluorescent sensor was made from commercially available fluorescent polymers coated onto glass beads, and its ability to discriminate explosives and explosive-related compounds in water was tested [85]. The chemometric study was carried out through principal component analysis (PCA) and linear discriminant analysis (LDA).

Quenching of room-temperature phosphorescence and enhancement of Rayleigh scattering based on Mn-doped ZnS QDs were used as a dual-recognition probe for TNT in water [86].

## 5. Other Organic Pollutants

2,6-Dinitrophenol (2,6-DNP) is one of the six possible dinitrophenol forms used in the synthesis of dyes, picric acid, picramic acid, wood preservatives, explosives and insecticides, and is a compound of environmental concern [87]. An optic fiber-based chemical sensor based on poly(vinyl chloride) containing a fluorescent curcumin moiety (FPVC) was developed by Wang *et al*. for the determination of 2,6-DNP in water [88]. FPVC extracts 2,6-DNP from the aqueous solution into the bulk membrane phase, leading to a significant fluorescence quenching of the curcumin moiety.

**Figure 3.**
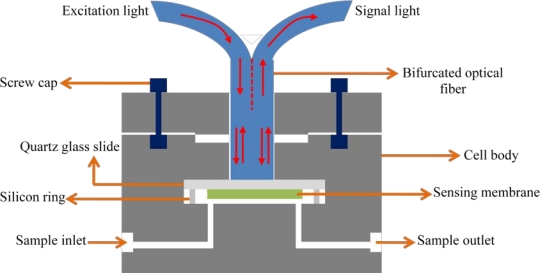
Schematic diagram of a bifurcated optical fiber flow cell arrangement.

The same group described bifurcated optical fiber chemical sensors (Figure 3) for the determination of bisphenol A (BPA), which is a chemical intermediate used in the synthesis of polycarbonate, epoxi, and unsaturated polyester-styrene resins and flame retardants [89,90]. One of the proposed methods is based on the formation of an inclusion complex between an insoluble β-cyclodextrin polymer and BPA, which enhances the luminescence of BPA. The other one monitors the fluorescence decrease of a pyrene/dimethyl-β-CD complex upon the addition of BPA, attributed to the displacement of pyrene by BPA. The developed sensors were successfully used for the determination of BPA in water samples and landfill leachates.

An optical inmunosensor to determine BPA in water samples was described [91]. This sensor is based on a solid-phase indirect immunoassay which takes place at an optical transducer chip, chemically modified with an analyte derivative. The labelled antibody (with a fluorescent trace) is bound to the transducer, and produces a fluorescent signal which can be correlated with the analyte concentration. The sensor surface can be regenerated allowing about 300 measurements with the same transducer, with a detection limit of 0.014 μg L^−1^.

**Figure 4.**
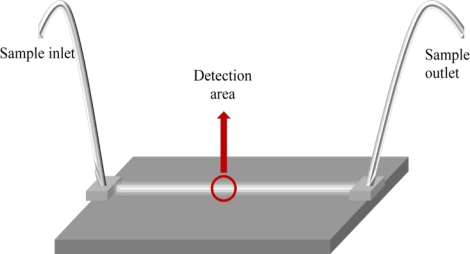
Schematic diagram of a single-flow immunosensor chip.

A micro-flow immunosensor chip (Figure 4) was constructed for quantifying coplanar polychlorinated biphenyls (Co-PBCs). The latter, although valuable compounds for many industrial applications, are persistent contaminants included in the highly toxic dioxine group [92]. Co-PBC antibodies immobilized in polyestyrene beads were introduced into the flow channel. The sample solution, mixed with both HRP and non-HRP conjugated antigens, was allowed to react in the flow channel. After the antigen-antibody reaction, a solution containing hydrogen peroxide and the fluorogenic substrate (10-acetyl-3,7-dihydroxyphenoxazine) produced a fluorescent dye (resorfin), and the signal was captured by a CCD camera of a fluorescence microscope. After the measurement of the fluorescence intensity, antigen–antibody complex immobilized beads were removed, and new antibody-immobilized beads were placed into the micro-flow channel for each repetitive measurement. The sensor allows to determine Co-PCB derivatives up to 0.1 part-per-trillion in 30 s, with a linear range from 0.1 to 1.0 μg mL^−1^.

Zhen *et al*. established a fluorescent probe for the qualitative detection of formaldehyde (a chemical widely used to manufacture building materials and household products) based on the signal produced by reaction of the analyte and the Nash reagent (ammonium acetate, acetic acid, acetyl acetone) embedded in silica gel beads [93]. The reagent consumption makes the probe unsuitable for continuous recording.

Tetracyclines (TCs) are considered as emergent pollutants [94], and their presence in food and water samples may affect the human health producing allergic reactions (even in low levels) and increased microbial antibiotic resistance caused by the daily intake. Traviesa Alvarez *et al*. have proposed a flow-through optosensor, based on the luminescence of Eu(III)-TCs complex retained on polymeric Amberlite XAD-4 particles packed in a flow-cell, to determinate four TCs (tetracycline, oxytetracycline, chlortetracycline and doxycycline) in bovine milk and water [95]. Recently, another flow-through fluorescence optosensor for TCs determination has been reported: the TC derivative included in CTAB micelles is retained onto the surface of Sephadex G-50 microbeads packed into the flow cell, followed by fluorescent detection of the TC derivative. The regeneration of the solid support is easily carried out employing deionized water. The limit of detection for TC in surface water samples was of 1.0 μg L^−1^ [96].

A fluorescence resonance energy quenching method using mercaptosuccinic acid-capped CdTe QDs immobilized on silica nanoparticles was developed for the quantification of 1,4-dihydroxybenzene (DHB) in water samples [97]. DHB is a benzene derivative widely used in dye, cosmetic, pesticide and pharmaceutical industries, and is considered a conspicuous environmental pollutant. In the proposed method, DHB molecules on the silica nanoparticles quench the fluorescence of the QDs, reaching a detection level as low as 2.4 × 10^−12^ mol L^−1^.

Sainz Gonzalo *et al*. [98,99] synthesized an MIP using toluene as template, which was implemented in a fluorescence optosensor for analyzing toluene, ethylbenzene and xylenes in river, tap and river water samples. The method allows to rapidly detect contaminated samples with a cut-off level of 700 μg mL^−1^ and 10 μg mL^−1^ for ethylbenzene and xylenes respectively.

Valero Navarro *et al*. developed a polyurethane-based magnetic MIP probe for the optical determination of 1-naphthylamine in tap and mineral water [100]. The method, with a limit of detection of 18 ng mL^−1^, allowed the determination of 1-naphthylamine in the presence of four structurally related compounds (2-naphthylamine, 1-naphthol, 2-naphthol and 1-naphthalenemethylamine).

Finally, a summary of all reviewed works is shown in Table 2.

## 6. Concluding Remarks

An important increase in activity for the development of luminescence sensors can be observed during the reviewed period. In general, optosensor methodologies are easily adaptable to green-chemistry principles, and therefore they offer a way to detect/quantify toxic pollutants without contaminating the environment which they want to preserve. Although measurements performed in batch mode are still applied, a significant increase in flow-injection approaches is noticed. The coupling of optosensors to second-order multivariate calibration brought about a significant increase in selectivity, allowing determinations in highly interferent media.

## Figures and Tables

**Table 1.** Techniques used for performing luminescence probe methodologies.

**Solid support**	**Brief description**	**Reference**
Plane surfaces	– Direct deposit of the sample over the sensor surface. The surface containing the analyte is placed in specially designed surface holder for measuring.	[15]
	– Solid-phase extraction via a syringe procedure. The surface containing the analyte is placed in specially designed surface holder for measuring.	[16]
	– Sensor films doped with a selected reagent in contact with the analyte solution contained in the reading cell.	[17]
Microbeads	Equilibrium is established between microbead active sites (with or without an auxiliary reagent) and the analyte. Beads are then transferred to a spectrofluorimetric cell or to a specially designed holding device (e.g., beads between two appropriately supported quartz sheets) for measuring.	[18]
Microbeads or plane surfaces	Sensor support (with or without an auxiliary immobilized reagent) in contact with the analyte solution. The measurement is carried out through an integrated optical fiber device.	[4,5]

**Table 2.** Luminescence sensors for organic pollutants in natural waters.

**Analyte**	**Configuration**	**Luminescence signal**	**Reagent/Solid support**	**LOD**	**Refs**
Benzo[*a*]pyrene	FTO	Phosphorescence	Non-ionic resin	12 ng mL^−1^	[52]
Fluoranthene	FTO	Phosphorescence	MIP	35 ng L^−1^	[53]
Benzo[*a*]pyrene	Probe	Fluorescence (F), phosphorescence (P)	Nylon membrane	0.14 ng L^−1^ (F), 5.9 ng L^−1^ (P)	[54]
Benzo[*a*]pyrene (BaP), dibenz[*a,h*]anthracene (DBA)	Probe	Fluorescence	Nylon membrane	Without interferents: 1.4 ng L^−1^ (BaP and DBA). With interferents: 5.7 ng L^−1^ (BaP), 4.1 ng L^−1^ (DBA)	[55]
Benzo[*a*]pyrene (BaP), dibenz[*a,h*]anthracene (DBA), chrysene (CHR), benzo[*b*]fluoranthene (BbF), benzo[*k*]fluoranthene (BkF), benz[*a*]anthracene (BaA)	FTO	Fluorescence	C_18_ silica gel	Without interferents: 4 ng L^−1^ (BaP), 69 ng L^−1^ (DBA), 25 ng L^−1^ (CHR), 3 ng L^−1^ (BbF), 4 ng L^−1^ (BkF), 22 ng L^−1^ (BaA). With interferents: 16 ng L^−1^ (BaP), 115 ng L^−1^ (DBA), 37 ng L^−1^ (CHR), 6 ng L^−1^ (BbF), 5 ng L^−1^ (BkF), 57 ng L^−1^ (BaA)	[56]
o-Phenylphenol (o-PP), fuberidazole (FBZ)	Multi-commutated FTO	Fluorescence	C_18_ silica gel	6.1 ng mL^−1^ (o-PP), 0.18 ng mL^−1^ (FBZ)	[60]
1-Naphthol (NP), o-phenylphenol (o-PP), thiabendazole (TBZ)	FTO	Fluorescence	C_18_ silica gel	μg L^−1^ levels	[61]
Thiabendazole	Probe	Phosphorescence	Nylon membrane	0.010 μg mL^−1^	[62]
Thiabendazole	FTO	Fluorescence	Nylon powder	2.8 ng mL^−1^	[31]
Thiabendazole	FTO	Phosphorescence	Nylon powder	4.5 ng mL^−1^	[32]
Carbendazim (MBC), thiabendazole (TBZ)	Probe	Fluorescence	C_18_ membrane	1 × 10^−3^ μg mL^−1^ (MBC) 2 × 10^−4^ μg mL^−1^ (TBZ)	[63]
Thiabendazole (TBZ), fuberidazol (FBZ)	FTO	Fluorescence	C_18_	Without interferents: 4 ng mL^−1^ (TBZ), 0.3 ng mL^−1^ (FBZ). With interferents: 17 ng mL^−1^ (TBZ), 1 ng mL^−1^ (FBZ)	[48]
Paraoxon (POX), Diisopropyl phosphorofluoridate (DFP)	Fiber-optic biosensor	Fluorescence	Enzyme conjugated with reporter anchored on the optical waveguide	Quantitative detection: 1–800 μM (POX), 2–400 μM (DFP)	[64]
Imidacloprid	FTO	PIF	C_18_ silica gel	1.8 ng mL^−1^ (IV = 640 μL)	[65]
Pentachlorophenol	Probe	Phosphorescence	MIP-Mn-doped ZnS QDs	86 nM	[66]
Atrazine (ATZ), diuron (DIU)	Flow-injection arrangement with detection through an optical fiber	Chemiluminescence	Photosystem II complex-enzyme immobilized on magnetic beads	3 × 10^−8^ M (ATZ), 1 × 10^−8^ M (DIU)	[67]
Atrazine, simazine, diuron, isoproturon and paraquat	FTO (biosensor)	Fluorescence	Algal strains immobilized in a polycarbonate membrane	Between 0.5 and 10 μg L^−1^	[68]
Simazine	FTO (immunosensor)	Fluorescence	Controlled-pore glass covalently bound to protein A packed in a reactor	1.3 ng L^−1^	[69]
Metsulfuron-methyl	FTO	Micellar-enhanced PIF	C_18_ silica	0.71 (IV = 300 μL), 0.14 ng mL^−1^ (IV = 1,000 μL)	[70]
Linuron	FTO	Micellar-enhanced PIF	C_18_ silica	0.13 μg mL^−1^	[71]
Isoproturon	FTO (immunosensor)	Fluorescence	Sol-gel glass doped with monoclonal antibody	9.7 ng L^−1^	[72]
1-Naphthaleneacetic	FTO	Phosphorescence	Amberlite XAD-7	1.2 ng mL^−1^	[73]
2-Naphthoxyacetic acid	FTO	Fluorescence (F), phosphorescence (P)	Amberlite XAD-7 (F) and silica gel (P)	2 ng mL^−1^ (F), 4.9 ng mL^−1^ (P)	[74]
Carbendazim (MBC), benomyl (BNM), carbofuran (CF)	FTO	Fluorescence	C_18_ silica	15 ng mL^−1^ (MBC), 35 ng mL^−1^ (BNM), 68 ng mL^−1^ (CF)	[75]
Thiabendazole (TBZ), metsulfuron (MET)	FTO	PIF	C_18_ silica	2.5 ng mL^−1^ (TBZ), 3.3 ng mL^−1^ (MET)	[76]
EA2192, VX, sarin, soman	Probe	Lanthanide-sensitized luminescence	MIP containing europium polymerized onto a fiber-optic	Using 15 min exposure times: 11 ng L^−1^ (EA2192), 21 ng L^−1^ (VX), 24 ng L^−1^ (sarin), 33 ng L^−1^ (soman)	[80]
2,4,6-Trinitrotoluene	Probe	Resonance energy transfer-amplifying fluorescence quenching	Covalently modified hybrid silica nanoparticles (assemble cheep and suspensions)	∼1 nM (nanoparticle-assemble cheep)	[83]
2,4-Dinitrotoluene (DNT), 2,4,6–trinitrotoluene (TNT)	Probe	Fluorescence quenching	QD labeled MIP microparticles	30.1 μM (DNT), 40.7 μM (TNT)	[84]
2,4,6–Trinitrotoluene	Probe	Phosphorescence quenching	Mn-doped ZnS QDs	0.8 nM	[86]
2,6–Dinitrophenol	Bifurcated optical fiber based flow optosensor	Fluorescence quenching	Plasticized PVC-curcumin moiety membrane	1.0 × 10^−6^ M	[88]
Bisphenol A	Bifurcated optical fiber based flow optosensor	Fluorescence	Plasticized PVC-β-CD polymer membrane	1.0 × 10^−6^ M	[89]
Bisphenol A	Bifurcated optical fiber based flow optosensor	Fluorescence quenching	pyrene/dimethyl-β-CD complex immobilized in a plasticized PVC membrane	7.0 × 10^−8^ M	[90]
Bisphenol A	Flow-through immunosensor	Fluorescence	Glass surface chip chemically modified with analyte derivative	0.014 μg mL^−1^	[91]
Coplanar polychlorinated biphenyl derivatives	FTO (immunosensor)	Fluorescence	Polydimethylsiloxane chip sealed on a glass substrate	Sensing range up to 0.1 ng L^−1^	[92]
Tetracycline, oxytetracycline, chlortetracycline, doxycycline	Flow-through optosensor	Phosphorescence	Amberlite XAD-4	Detection range: 0.2–11.6 nM (cut-off level = 4 nM)	[95]
Tetracycline	FTO	Fluorescence	Sephadex G-50	1.0 μg L^−1^	[96]
1,4-Dihydroxybenzene	Probe	Fluorescence resonance energy quenching	Mercaptosuccinic acid-capped CdTe quatum dots immobilized on silica particles	2.4 × 10^−12^ M	[97]
Toluene, ethylbenzene, xylenes	FTO	Fluorescence	MIP	Range of concentrations used in the screening test: 0.5–2 μg mL^−1^ (toluene), 0.5–3 μg mL^−1^ (ethylbenzene), 1–20 μg mL^−1^ (xylenes)	[98]
Xylenes	FTO	Fluorescence	MIP	Range of concentrations used for each isomer in the screening test: 3–20 μg mL^−1^	[99]
1-Naphthylamine	Probe	Fluorescence	Polyurethane based magnetic-MIP	18 ng mL^−1^	[100]

CD: cyclodextrin; FTO: flow-through optosensor; IV: injection volume; MIP: molecularly-imprinted polymers; PIF: photochemically induced fluorescence; PVC: poly(vinyl chloride); QDs: quantum dots.
